# *Clostridioides difficile* infection surveillance in intensive care units and oncology wards using machine learning

**DOI:** 10.1017/ice.2023.54

**Published:** 2023-11

**Authors:** Erkin Ötleş, Emily A. Balczewski, Micah Keidan, Jeeheh Oh, Alieysa Patel, Vincent B. Young, Krishna Rao, Jenna Wiens

**Affiliations:** 1 Medical Scientist Training Program, University of Michigan Medical School, Ann Arbor, Michigan; 2 Department of Industrial & Operations Engineering, College of Engineering, University of Michigan, Ann Arbor, Michigan; 3 Department of Computational Medicine and Bioinformatics, University of Michigan, Ann Arbor, Michigan; 4 Division of Infectious Diseases, Department of Internal Medicine, University of Michigan Medical School, Ann Arbor, Michigan; 5 Department of Electrical Engineering and Computer Science, College of Engineering, University of Michigan, Ann Arbor, Michigan; 6 Department of Pathology, University of Michigan Medical School, Ann Arbor, Michigan; 7 Department of Microbiology and Immunology, University of Michigan Medical School, Ann Arbor, Michigan

## Abstract

**Objective:**

Screening individuals admitted to the hospital for *Clostridioides difficile* presents opportunities to limit transmission and hospital-onset *C. difficile* infection (HO-CDI). However, detection from rectal swabs is resource intensive. In contrast, machine learning (ML) models may accurately assess patient risk without significant resource usage. In this study, we compared the effectiveness of swab surveillance to daily risk estimates produced by an ML model to identify patients who will likely develop HO-CDI in the intensive care unit (ICU) setting.

**Design:**

A prospective cohort study was conducted with patient carriage of toxigenic *C. difficile* identified by rectal swabs analyzed by anaerobic culture and polymerase chain reaction (PCR). A previously validated ML model using electronic health record data generated daily risk of HO-CDI for every patient. Swab results and risk predictions were compared to the eventual HO-CDI status.

**Patients:**

Adult inpatient admissions taking place in University of Michigan Hospitals’ medical and surgical intensive care units and oncology wards between June 6th and October 8th, 2020.

**Results:**

In total, 2,979 admissions, representing 2,044 patients, were observed over the course of the study period, with 39 admissions developing HO-CDIs. Swab surveillance identified 9 true-positive and 87 false-positive HO-CDIs. The ML model identified 9 true-positive and 226 false-positive HO-CDIs; 8 of the true-positives identified by the model differed from those identified by the swab surveillance.

**Conclusion:**

With limited resources, an ML model identified the same number of HO-CDI admissions as swab-based surveillance, though it generated more false-positives. The patients identified by the ML model were not yet colonized with *C. difficile*. Additionally, the ML model identifies at-risk admissions before disease onset, providing opportunities for prevention.

Hospital-onset *Clostridioides difficile* infections (HO-CDIs) are the most common nosocomial diarrheal illness, leading to significant morbidity and mortality among hospitalized patients.^1,2^ Early identification of HO-CDI could help decrease risks of morbidity and mortality.^3,4^ In particular, screening and isolating individuals colonized with *C. difficile* could reduce the number of HO-CDI cases.^5,6^ However, due to a lack of strong evidence, the IDSA does not recommend the implementation of screening and isolation of asymptomatic carriers.^7^

Screening is primarily conducted by detecting the presence of toxigenic *C. difficile* in rectal swabs via anaerobic culture or directly with polymerase chain reaction (PCR). Swabs require collection and testing materials (ie, swabs, media, and PCR reagents), and swab collection imposes an additional workflow burden on busy nursing staff. Thus, swab-based surveillance is invasive and resource intensive. Additionally, the collection is invasive because the swabbing process collects material from the rectal mucosa.

In contrast, machine learning (ML) patient-risk stratification models embedded in electronic health record (EHR) systems assess patient risk noninvasively and without interrupting existing workflows. Several ML models exist for identifying patients at risk of HO-CDI.^8–10^ To date, researchers have extensively validated an approach for predicting HO-CDI using ML that generates daily risk estimates based on the contents of the EHR.^8,11–13^ However, how to best use these risk estimates to target interventions remains unclear. Specifically, the extent to which patients identified as high-risk by the algorithm are already colonized with *C. difficile* is unknown. Such knowledge could inform model use.

We need to understand whether and how the populations identified by rectal swab surveillance and ML approaches differ. To address these gaps, we sought to answer the following questions: Does an ML approach have comparable performance in the early detection of HO-CDI when compared to rectal swab surveillance? And do both approaches identify the same populations? In answering these questions, we compared the populations identified by rectal swab surveillance versus a validated ML model identifying those at risk for HO-CDI among a population of intensive care unit (ICU) and oncology patient admissions.

## Methods

### Outcome

The outcome of interest in this study was primary, nonrecurrent hospital-onset CDI (HO-CDI), defined by the Centers for Disease Control and Prevention (CDC) as a positive clinical laboratory test >3 days after admission to a healthcare facility and not occurring within 8 weeks after successful treatment of a prior CDI episode.^14^ Prediction of the outcome was analyzed at the level of each admission. During the study period, the University of Michigan Hospitals used a 2-step, in-house clinical testing protocol for CDI.^15,16^ Briefly, patients with new-onset diarrhea and clinical suspicion of CDI first underwent testing via enzyme immunoassay (EIA) for glutamate dehydrogenase (GDH) antigen and *C. difficile* toxins TcdA and TcdB (C. Diff Quik Check Complete, TechLab, Blacksburg, VA), the combination of which yielded good sensitivity.^17^ If the results for GDH and toxins TcdA and TcdB were discordant, a PCR for toxin B determined the final test result reported to the clinician.

### Study cohort

As part of a pre-existing, separate, and ongoing VRE surveillance program at our hospital, patients aged 18 years or older admitted to University of Michigan Hospital ICUs and oncology wards between June 6 2020 and October 8, 2020, had rectal swabs collected on admission to the unit, weekly, and at discharge from the unit. Our study cohort included all admissions that had 1 or more swabs collected and had ML model risk estimates calculated. We excluded admissions in which the outcome of primary, nonrecurrent HO-CDI could not be met, admissions with inpatient stays <3 calendar days, and admissions who instead met criteria for community-onset CDI by positive clinical CDI testing the first 2 calendar days of admission or in the 14 calendar days before admission. Additionally, we excluded admissions who had successful treatment in the 8 weeks prior to admission date and who had rectal swabs collected after clinical CDI tests.

The study was approved by the institutional review board of the University of Michigan Medical School (no. HUM00147185 and no. HUM00170413).

### Swabs

Rectal swabs were collected as a part of the University of Michigan Hospitals’ VRE surveillance protocol. The nursing staff was instructed to either pass the flocked-E swab through the anal verge or dip it in a fresh stool sample. Swabs were placed in containers with Amie’s media, and 100 μL aliquots were frozen and stored at −80° C for analysis in batches 6–12 months later. Aliquots were anaerobically cultured for 24 hours in sodium taurocholate cycloserine-cefoxitin-fructose (TCCFB) media; any growth was then plated on commercially available CHROMagar plates with conditions designed for selective growth of *C. difficile*, and single colonies were used for subsequent confirmation of taxonomy and toxigenicity using *C. difficile*-specific 16S, TcdB, and TcdA PCR assays.^18,19^

### Model application

The risk of HO-CDI was calculated daily using a previously validated EHR-based ML modeling approach.^11–13^ Risk estimates, based on the contents of the EHR were calculated daily for each patient admission starting on the day 3 of the admission and until the day of discharge or when criteria for HO-CDI were first met.

Briefly, the ML model was an L2-regularized logistic regression model trained to stratify patients at risk of HO-CDI. The model takes as input data pertaining to patient demographics (sex, age, race), daily in-hospital locations (current unit), and daily clinical characteristics (vital signs, medications, number of prior hospitalizations, etc) and outputs a value between 0 and 1 that corresponds to an estimate of the patient’s risk of developing CDI during the remainder of the hospitalization. These estimates are generated daily and updated over time as the clinical characteristics of in-hospital locations change.^20^ Oh and Makar et al^11^ describe the initial model development in detail.

### Analysis

Swab results and model risk estimates were independently assessed as surveillance tools for HO-CDI. Both were assessed at the level of each admission (also known as an encounter). This is different from analysis at the patient level because a single patient may have multiple admissions. An admission was defined as the contiguous unit of time between hospital admission and discharge. Over the course of the admission, 1 or more swabs were collected and several ML model risk estimates were generated. We only included swabs and risk estimates available prior to clinical CDI testing. For example, if a clinical CDI test was ordered on day 5 of the patient admission only swab collected on day 1 and model risk estimates from days 1–4 would be considered.

To assess the performance of both approaches, we evaluate multiple observations (ie, multiple swabs or multiple model risk estimates) at the level of the admission. For a given admission, if any of the swabs (collected prior to the clinical CDI test) resulted positive, the swab score for that admission was 1. Similarly, we used the maximum risk estimate (prior to clinical CDI test), as the “risk score.” To binarize the numerical risk score generated by the ML model, we selected a threshold that yielded a sensitivity (ie, the likelihood deeming a patient ‘high-risk’ given that a patient admission will experience HO-CDI) equivalent to that of the swab-based results. These admission-level binary “scores” ensure consistent representation and fair comparison. This evaluation is based on the idea that once a patient exceeds some risk threshold or their swab returns positive one would intervene.

For our primary analysis, we assessed binary classification performance in the form of confusion matrices, accuracy, specificity, positive predictive value (PPV), negative predictive value (NPV), and F1 score (the harmonic mean of precision and recall) to compare the 2 methods. We also examined the patient population identified by each “surveillance” approach.

In addition to analyzing each “surveillance” approach individually, we also examined 2 approaches that combine swab and model information. The first approach, which we refer to as “model AND swab,” flagged a patient as high risk if the swab indicated colonization and the model produced at least 1 risk estimate above the threshold. The second approach, “model OR swab,” flagged patients as high risk if either of the above criteria were met. In a supplementary analysis, we also examined the receiver operating characteristics of the 2 approaches.

## Results

**Fig. 1.**
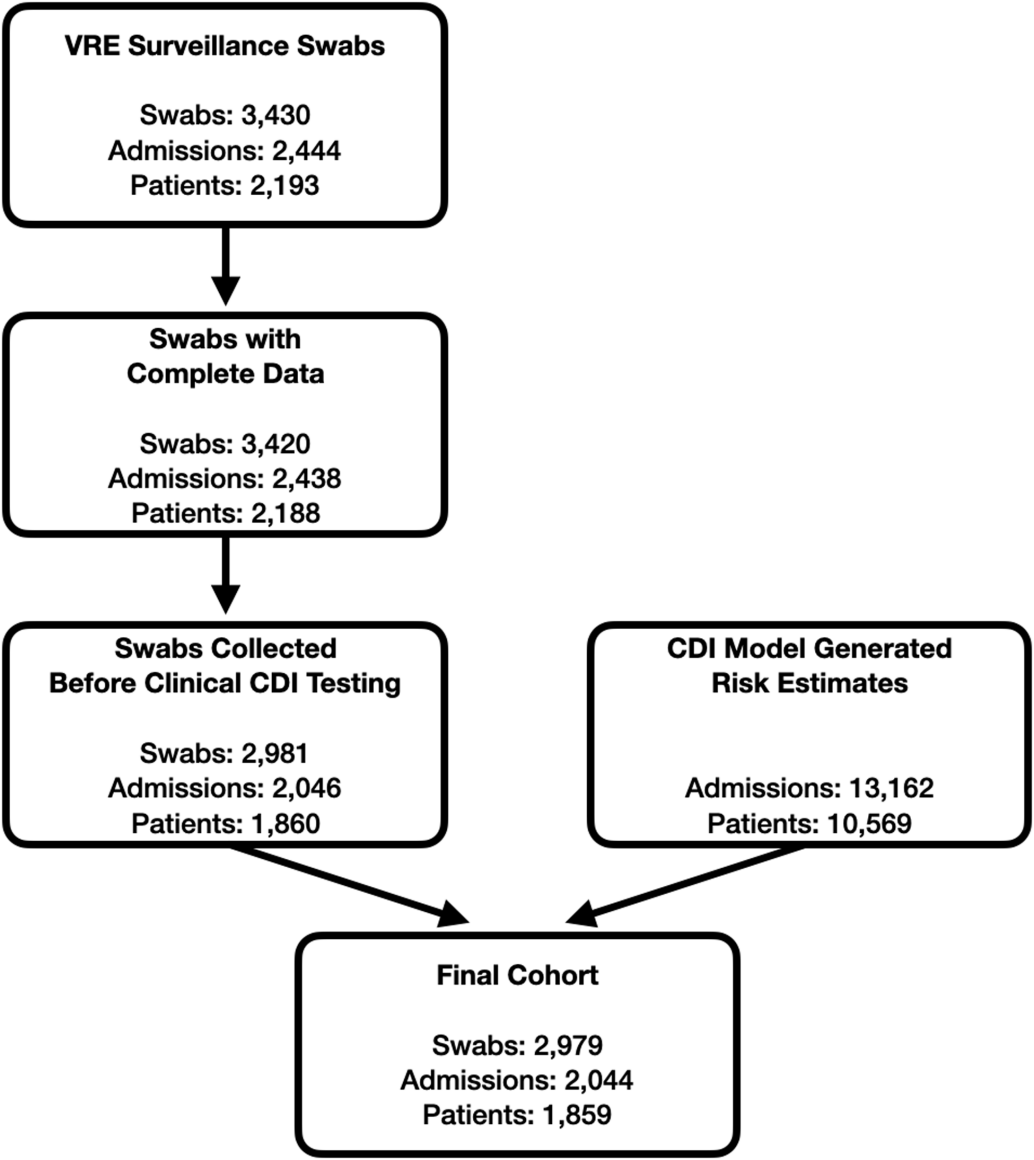
Cohort development. A patient admission needed to have at least 1 swab collected and 1 or more machine learning (ML) model risk estimates generated before clinical *Clostridioides difficile* infection (CDI) testing to be included in the study cohort. Thus, swabs missing culture information and swabs collected after the model stopped evaluating a patient (due to clinical CDI testing) were excluded.

Our final study cohort included 2,044 admissions, representing 2,979 unique swabs and 1,859 patients; 39 (1.9%) admissions met the primary outcome (Fig. 1). These admissions all had at least 1 swab collected and 1 or more model risk estimates generated before clinical CDI testing was conducted. Reflecting a critical care population, the median length of stay was 9 days (IQR, 5–17 days); 553 admissions (27.1%) had a prior admission in the previous 90 days (Table 1). On average, patients had 1 swab collected per admission prior to HO-CDI (IQR, 1–2) because swabbing only occurred upon admission to the unit, weekly and upon discharge. In comparison, patients had, on average, 6 model risk estimates (IQR, 3–14) because risk estimates were produced daily from calendar day 3 of the admission onward, regardless of in-hospital location.

**Table 1.** Demographics, Clinical Characteristics, Outcomes, and Surveillance Characteristics of the Final Study Cohort

Patient Demographics	Total (n=2,044), No. (%)^a^
Sex, female (per EHR)	841 (41.1)
Age at admission, median y [IQR]	61 [50–70]
**Race**	
Asian	47 (2.3)
Black	211 (10.3)
White	1,669 (81.7)
Other	94 (4.6)
Missing	23 (1.1)
**Ethnicity**	
Hispanic or Latino	1,770 (86.6)
Not Hispanic nor Latino	36 (1.8)
Other	238 (11.6)
Length of stay, median d [IQR]	9 [5–17]
**Clinical characteristics**	
90-d history	
Prior University of Michigan hospital admission	553 (27.1)
Immunosuppressants prior to admission	63 (3.1)
Gastric-acid suppressants prior to admission	188 (9.2)
Antibiotics prior to admission	373 (18.3)
Enteral feeding prior to admission	0 (0)
**Index admission**	
Immunosuppressant usage	229 (11.2)
Gastric-acid suppressant usage	898 (43.9)
Antibiotic usage	1805 (88.3)
Enteral feeding usage	11 (0.5)
**Admissions with prior CDI history**	
CDI within last 90 d	11 (0.5)
CDI within last year	26 (1.3)
Outcome surveillance	
Clinical diagnosis of CDI	39 (1.9)
CDI colonization pressure cases/10,000 PD	15.7
**Rectal swabs**	
Toxigenic *C. difficile* cultured from rectal swab	96 (4.7)
No. collected per admission, median [IQR]	1 [1–2]
**Model**	
Risk estimates, median [IQR]	0.5 [0.4–0.7]
No. of scores per admission, median [IQR]	6 [3–14]

Note. EHR, electronic health record; IQR, interquartile range; PD, patient days; CDI, *Clostridioides difficile* infection.

a Units unless otherwise specified.

**Fig. 2.**
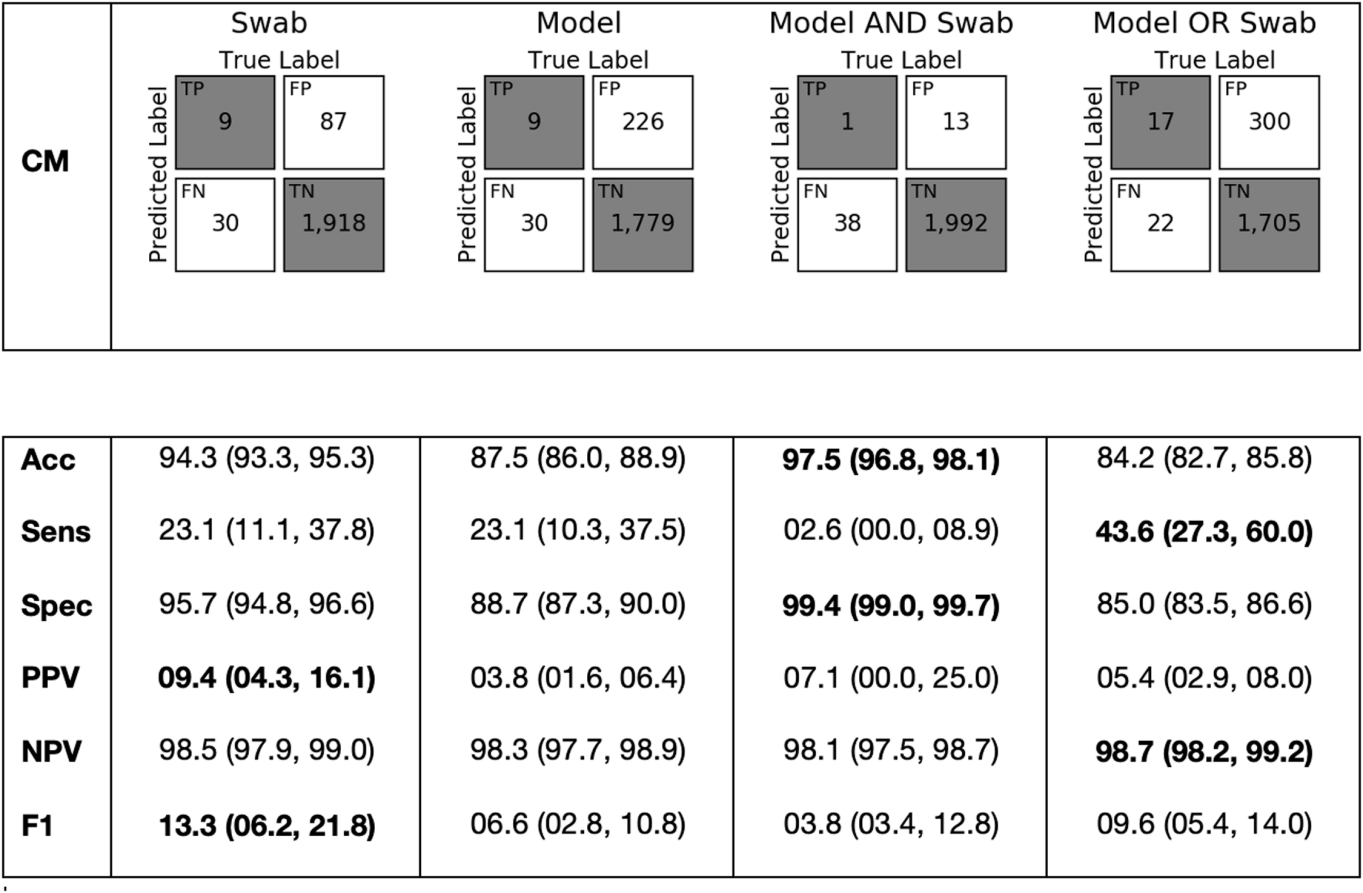
Performance of swab, model, and combinations. Top panel shows the confusion matrices (CM) of each approach. Bottom panel shows binary performance measures, accuracy (Acc), sensitivity (Sens), specificity (Spec), positive predictive value (PPV), negative predictive value (NPV), and F1 score.

Swab surveillance identified 96 admissions (4.7%) as colonized with *C. difficile*, resulting in sensitivity of 23.1% (95% confidence interval [CI], 11.1%– 37.8%), accuracy of 94.3% (95% CI, 93.3%–95.3%), specificity of 95.7% (95% CI, 94.8%–96.6%), PPV of 9.4% (95% CI, 4.3%–16.1%), NPV of 98.5% (95% CI, 97.9%–99.0%), and F1 of 13.3% (95% CI, 6.2%–21.8%). Holding sensitivity equal at 23.1% (95% CI, 10.3%–37.5%) to the swabs yielded a risk estimate threshold of 0.809 for the ML model. At this threshold, the model identified 235 admissions (11.5%) as high-risk, yielding accuracy of 87.5% (95% CI, 86.0%–88.9%), specificity of 88.7% (95% CI, 87.3%–90.0%), PPV of 3.8% (95% CI, 1.6%–6.4%), NPV of 98.3% (95% CI, 97.7%–98.9%), and F1 of 6.6% (95% CI, 2.8%–10.8%). These findings are summarized in Figure 2. At this sensitivity level, the swab-based approach demonstrated superior accuracy, specificity, and PPV. There was no statistical difference between the NPV and F1 of the 2 approaches. Although both approaches correctly identified 9 of 39 HO-CDI patients, these were not the same patients, overlapping in only a single case.

We also evaluated the utility of the combination of swabs and the ML model. Across all approaches, the model-AND-swab approach yielded the highest accuracy of 97.5% (95% CI, 96.8%–98.1%) and specificity of 99.4% (95% CI, 99.0%–99.7%). The model-OR-swab combination yielded the highest sensitivity of 43.6% (95% CI, 27.3%–60.0%) and NPV of 98.7% (95% CI, 98.2%–99.2%). Again, these results differed because the true-positive results identified by the swab versus the ML model only overlapped in the case of 1 admission.

From our secondary analysis (see supplemental material), the ML approach without a threshold outperformed the swab in terms of AUROC (swab, 59.2%; model, 73.4%).

## Discussion

In this study, we compared the HO-CDI prediction capacity of 2 surveillance approaches: swab surveillance and daily risk estimates produced by an EHR-based ML model. We observed a HO-CDI incidence rate of 1.9%, which is slightly higher than the overall incidence rate across the University of Michigan Hospitals in 2020. This difference was expected because the units included in the study admit a greater number of critically ill patients who are susceptible to disease. The swab-based approach identified 4.7% of admissions as having toxigenic *C. difficile* and had a sensitivity of 23.1%. When holding the sensitivity of the ML approach equal to that of the swab approach, the model flagged many more admissions (ie, 11.5%) as high risk.

Overall, the swab approach yielded significantly fewer false-positive results and had slightly better binary outcome prediction measures: accuracy, specificity, PPV, NPV, and F1 score. Both approaches correctly identified the same number of HO-CDI patients (n = 9); however, they identified different populations, with each approach identifying 8 patients that the other did not. Thus, the combination of these 2 approaches presents an opportunity to improve surveillance.

Although the ML model-based surveillance system had slightly worse performance in binary outcome prediction measures, it was able to identify a subset of patients at risk for HO-CDI who were not identified as colonized with *C. difficile*. Compared to the resource-intensive swab-based approach, the ML model scales easily to all units in a hospital and produces scores daily rather than only weekly. Moreover, the ML model does not require rectal swab collection across large populations of hospitalized patients, and it does not depend on the implementation of specialized laboratory tests that most medical centers are not equipped to run. The development and integration of EHR-based ML models are not without costs. Significant financial costs are associated with the development and implementation of this model due to the amount of developer time needed. Additionally, depending on the threshold selected, the model may generate many false-positive alerts, leading to a risk of alert fatigue.

However, once implemented, a model-based approach costs very little to apply. This means that the up-front fixed costs of a CDI model can be amortized over many uses. For example, we expect the CDI model implemented at the University of Michigan Hospitals to produce >255,000 risk estimates over the course of a year. These daily risk estimates are available for nearly every admission, which is a major advantage compared to rectal swabbing, which occurs at certain predetermined time points and locations within the hospitals. Depending on the course of the patient’s admission, they may not have a rectal swab collected before they have a clinical CDI test conducted. For example, in this study, we had 392 admissions who had their first rectal swabs collected after a clinical CDI test had been conducted. Additionally, in the same time that 2,979 rectal swabs were collected for the 2,044 admissions, the ML model had produced 13,162 daily risk estimates. Combined, the lightweight resource footprint and scalability make the ML model an attractive adjunct, or even standalone, infection surveillance system.

Interestingly, only 1 patient who eventually went on to acquire HO-CDI was flagged by both surveillance approaches as being high risk. The small overlap in true positives between swab and ML predictions suggests that these two approaches identify different subgroups of high-risk patients. Although swab surveillance only identifies patients already colonized with CDI, the ML model identifies susceptible patients prior to colonization with the pathogen. Many of the covariates used as input to the model pertain to susceptibility (eg, medications that suppress the immune system or disrupt the gut microbiome). Since swab surveillance cannot identify patients who are not already colonized, there might be an additional advantage of the ML models in their ability to identify patients before colonization. This ability could be used to target infection prevention and control efforts (eg, handwashing with soap and water), preventing colonization and subsequent infection. Moreover, the fact that the model starts producing estimates early in the patient’s admission prior to admission to the ICU provides more time to target prevention efforts.

Patients identified by the ML approach may be candidates for interventions that protect them from potential exposures, such as use of soap and water prior to patient contact, which reduces risk of exposure to *C. difficile* spores, and that stop or de-escalate treatments that increase risk of colonization and subsequent CDI, such as broad-spectrum antibiotics and proton pump inhibitors.^21^ For example, patient risk of CDI could be integrated into existing pharmacy workflows, improving antimicrobial stewardship efforts. Applied in a targeted manner, such interventions could help reduce *C. difficile* incidence and onward transmission. Here, the precise definition of highest risk (ie, the risk threshold) depends on resource constraints. For example, a threshold hold associated with the 95th percentile of risk corresponds to intervening in ∼5% of patient encounters.

The high number of false-positive results in the ML model-based approach drove its relatively poor performance. This is problematic for several reasons. First, it increases the overall number of alerts and interventions, potentially increasing the overall time and costs associated with surveillance and intervention. Second, the large number of false-positive results may induce alert fatigue and mistrust of the surveillance system, leading to decreased user adoption.^22^

Unlike swab surveillance, which returns a binary result instead of a continuous one, the high-risk threshold may be tuned to obtain a more favorable balance of true-positive results versus false-positive results. Because the model produces a continuous estimate of risk, by increasing the threshold at which an individual is deemed ‘high risk,’ the model can trade sensitivity for greater specificity (ie, reducing the number of false positives) (see Supplementary Fig. S1). Additionally, the probability of a true-positive alert can be improved by limiting the use of the model to units with a higher incidence of CDI. Ultimately study of user behavior and response to the model should guide such implementation efforts.

Given the high cost of managing CDI relative to surveillance, surveillance-based interventions may prove cost-effective.^23^ The relatively lower cost and flexibility of the ML approach may provide additional value in infection prevention and early identification of CDI in the hospital setting, as long as the costs of false-positive results do not outweigh the benefits.

Importantly, our results may not generalize to other hospital systems or patient populations, given that these data were only gathered at the University of Michigan Hospitals’ adult ICU and oncology wards. Additionally, CDI rates may substantially increase and decrease seasonally,^24^ and our study only examined 4 months over the summer and early fall. These methods may perform differently when rates of CDI are higher or lower than in our cohort. Moreover, the results presented were evaluated at the level of an admission. The 23.1% sensitivity of the swab-based approach should not interpreted as the sensitivity of an individual swab. Finally, these data were obtained during the COVID-19 pandemic, which may have affected our cohort’s baseline patient characteristics and *C. difficile* hospital transmission dynamics.^25^

Despite these limitations, our study motivates further investigation into the benefits of using EHR-based ML models for an accurate, low-cost, and resource-sparing estimate of HO-CDI risk. To this end, we are exploring ways in which we can use ML-based risk estimates to guide infection prevention efforts to reduce CDI incidence at the University of Michigan. In addition, our results motivate further study of both swab-based and ML model-based surveillance of CDI. Furthermore, the approaches are not mutually exclusive and a mixed strategy using both rectal surveillance and ML-based risk estimates could be employed to reduce the burden of CDI in hospitals.

## Supplementary material

For supplementary material accompanying this paper visit https://doi.org/10.1017/ice.2023.54. click here to view supplementary material
