# Supplementary material for: Clostridioides difficile infection surveillance in intensive care units and oncology wards using machine learning
Source: Infect Control Hosp Epidemiol. 2023 Apr 24;44(11):1776–81. doi: 10.1017/ice.2023.54 (PMC10665879; doi:10.1017/ice.2023.54)
Supplement: Supplementary file 1 [file S0899823X23000545sup001.docx]

**Supplemental Material**

**Methods**

A secondary analysis was conducted regarding the discriminative performance of both approaches. We measured the discriminative performance in terms of the area under the receiver operating characteristics curve (AUROC). For this analysis, we employed the maximum observed risk estimate produced for each patient (*i.e.*, the threshold is not employed). In addition to the AUROC, we plotted the ROC curve, the precision recall (PR) curve, and report the area under it (AUPR). We computed 95% empirical bootstrap confidence intervals (CI) for the primary and secondary analyses using 1,000-fold patient-encounter resampled bootstraps.

Finally, we plotted the performance of the swabs and model for four encounters representing every combination of swab correct/incorrect and model correct/incorrect. These encounters were randomly selected from the population of encounters that met the given swab-model combination.

**Results**

Our secondary analysis shows that using the swabs to detect HO-CDI had an AUROC of 0.592 (95% CI: 0.474, 0.692), whereas the model risk estimates (without a threshold applied) had an AUROC of 0.734 (0.625, 0.829). **Figure S1** shows ROC and PR curves for the swabs and the model. Moreover, **Figure S2** shows the timelines for four encounters representing every combination of swab correct/incorrect and model correct/incorrect.

**
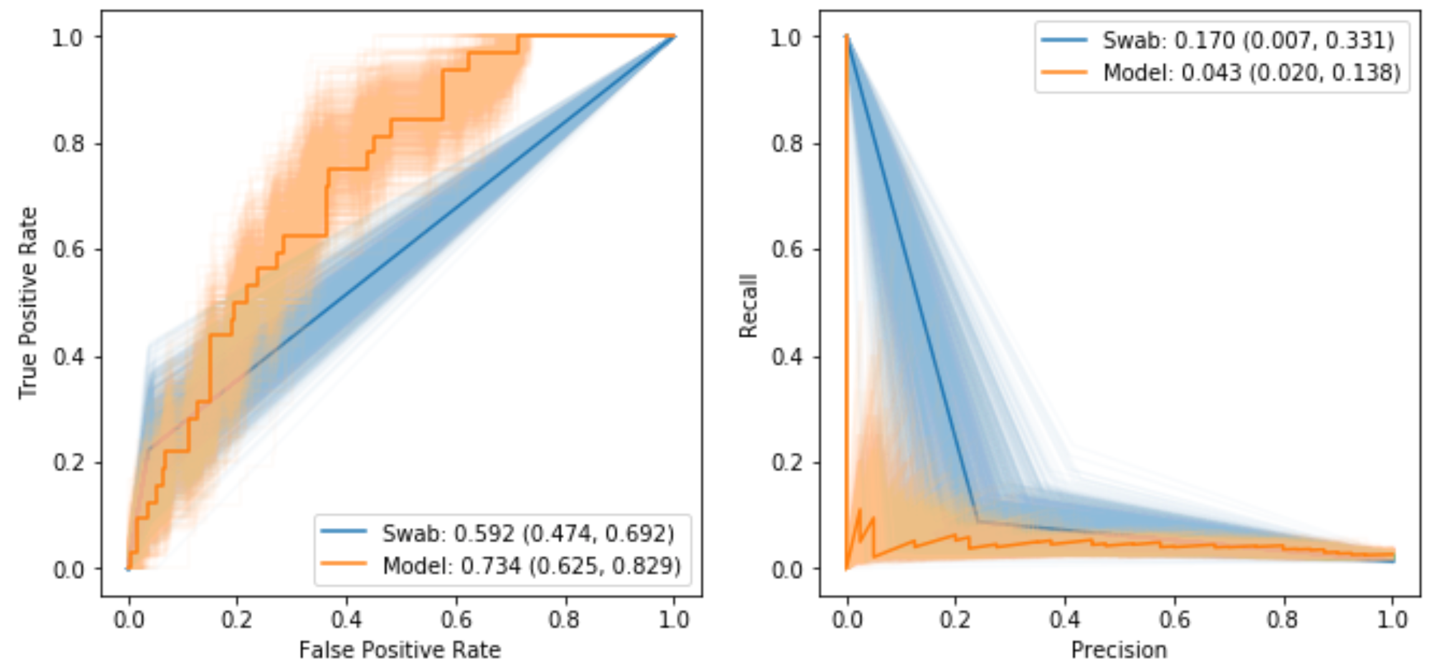
**

Figure S1: AUROC and AUPR of Swab and Model Based Approaches.


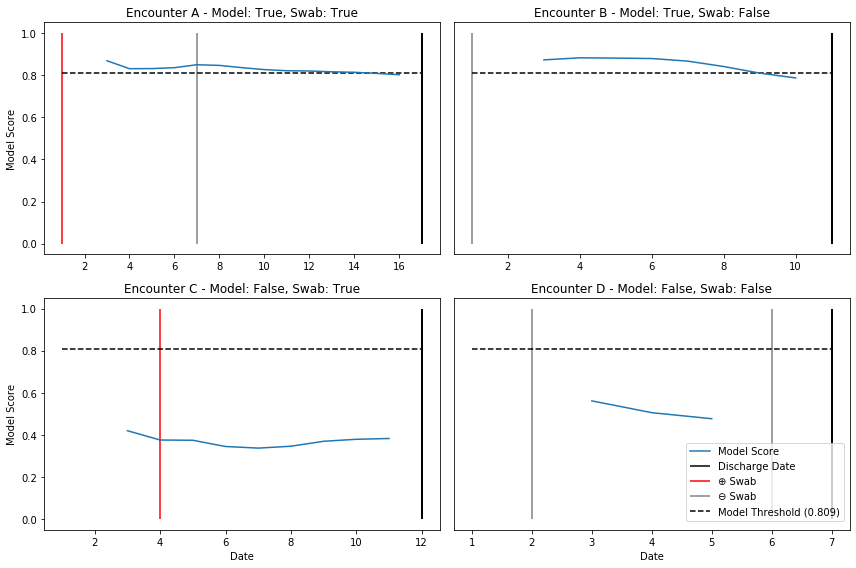


Figure 2: Example Encounter Timelines.
